# Ectopic Lymphoid Structures Support Ongoing Production of Class-Switched Autoantibodies in Rheumatoid Synovium

**DOI:** 10.1371/journal.pmed.0060001

**Published:** 2009-01-13

**Authors:** Frances Humby, Michele Bombardieri, Antonio Manzo, Stephen Kelly, Mark C Blades, Bruce Kirkham, Jo Spencer, Costantino Pitzalis

**Affiliations:** 1 William Harvey Research Institute, Barts and the London School of Medicine, Charterhouse Square, London, United Kingdom; 2 Department of Rheumatology, Kings College London, Guy's Hospital, St Thomas Street, London, United Kingdom; 3 Division of Infection, Immunity and Inflammatory Diseases, Kings College London, Guy's Hospital, St Thomas Street, London, United Kingdom; Leiden University Medical Centre, The Netherlands

## Abstract

**Background:**

Follicular structures resembling germinal centres (GCs) that are characterized by follicular dendritic cell (FDC) networks have long been recognized in chronically inflamed tissues in autoimmune diseases, including the synovium of rheumatoid arthritis (RA). However, it is debated whether these ectopic structures promote autoimmunity and chronic inflammation driving the production of pathogenic autoantibodies. Anti-citrullinated protein/peptide antibodies (ACPA) are highly specific markers of RA, predict a poor prognosis, and have been suggested to be pathogenic. Therefore, the main study objectives were to determine whether ectopic lymphoid structures in RA synovium: (i) express activation-induced cytidine deaminase (AID), the enzyme required for somatic hypermutation and class-switch recombination (CSR) of Ig genes; (ii) support ongoing CSR and ACPA production; and (iii) remain functional in a RA/severe combined immunodeficiency (SCID) chimera model devoid of new immune cell influx into the synovium.

**Methods and Findings:**

Using immunohistochemistry (IHC) and quantitative Taqman real-time PCR (QT-PCR) in synovial tissue from 55 patients with RA, we demonstrated that FDC+ structures invariably expressed AID with a distribution resembling secondary lymphoid organs. Further, AID+/CD21+ follicular structures were surrounded by ACPA+/CD138+ plasma cells, as demonstrated by immune reactivity to citrullinated fibrinogen. Moreover, we identified a novel subset of synovial AID+/CD20+ B cells outside GCs resembling interfollicular large B cells. In order to gain direct functional evidence that AID+ structures support CSR and in situ manufacturing of class-switched ACPA, 34 SCID mice were transplanted with RA synovium and humanely killed at 4 wk for harvesting of transplants and sera. Persistent expression of AID and Iγ-Cμ circular transcripts (identifying ongoing IgM-IgG class-switching) was observed in synovial grafts expressing FDCs/CD21L. Furthermore, synovial mRNA levels of AID were closely associated with circulating human IgG ACPA in mouse sera. Finally, the survival and proliferation of functional B cell niches was associated with persistent overexpression of genes regulating ectopic lymphoneogenesis.

**Conclusions:**

Our demonstration that FDC+ follicular units invariably express AID and are surrounded by ACPA-producing plasma cells provides strong evidence that ectopic lymphoid structures in the RA synovium are functional and support autoantibody production. This concept is further confirmed by evidence of sustained AID expression, B cell proliferation, ongoing CSR, and production of human IgG ACPA from GC+ synovial tissue transplanted into SCID mice, independently of new B cell influx from the systemic circulation. These data identify AID as a potential therapeutic target in RA and suggest that survival of functional synovial B cell niches may profoundly influence chronic inflammation, autoimmunity, and response to B cell–depleting therapies.

## Abstract 2

Costantino Pitzalis and colleagues show that lymphoid structures in synovial tissue of patients with rheumatoid arthritis support production of anti-citrullinated peptide antibodies, which continues following transplantation into SCID mice.

## Editors' Summary

**Background.:**

More than 1 million people in the United States have rheumatoid arthritis, an “autoimmune” condition that affects the joints. Normally, the immune system provides protection against infection by responding to foreign antigens (molecules that are unique to invading organisms) while ignoring self-antigens present in the body's own tissues. In autoimmune diseases, this ability to discriminate between self and non-self fails for unknown reasons and the immune system begins to attack human tissues. In rheumatoid arthritis, the lining of the joints (the synovium) is attacked, it becomes inflamed and thickened, and chemicals are released that damage all the tissues in the joint. Eventually, the joint may become so scarred that movement is no longer possible. Rheumatoid arthritis usually starts in the small joints in the hands and feet, but larger joints and other tissues (including the heart and blood vessels) can be affected. Its symptoms, which tend to fluctuate, include early morning joint pain, swelling, and stiffness, and feeling generally unwell. Although the disease is not always easy to diagnose, the immune systems of many people with rheumatoid arthritis make “anti-citrullinated protein/peptide antibodies” (ACPA). These “autoantibodies” (which some experts believe can contribute to the joint damage in rheumatoid arthritis) recognize self-proteins that contain the unusual amino acid citrulline, and their detection on blood tests can help make the diagnosis. Although there is no cure for rheumatoid arthritis, the recently developed biologic drugs, often used together with the more traditional disease-modifying therapies, are able to halt its progression by specifically blocking the chemicals that cause joint damage. Painkillers and nonsteroidal anti-inflammatory drugs can reduce its symptoms, and badly damaged joints can sometimes be surgically replaced.

**Why Was This Study Done?:**

Before scientists can develop a cure for rheumatoid arthritis, they need to know how and why autoantibodies are made that attack the joints in this common and disabling disease. B cells, the immune system cells that make antibodies, mature in structures known as “germinal centers” in the spleen and lymph nodes. In the germinal centers, immature B cells are exposed to antigens and undergo two genetic processes called “somatic hypermutation” and “class-switch recombination” that ensure that each B cell makes an antibody that sticks as tightly as possible to just one antigen. The B cells then multiply and enter the bloodstream where they help to deal with infections. Interestingly, the inflamed synovium of many patients with rheumatoid arthritis contains structures that resemble germinal centers. Could these ectopic (misplaced) lymphoid structures, which are characterized by networks of immune system cells called follicular dendritic cells (FDCs), promote autoimmunity and long-term inflammation by driving the production of autoantibodies within the joint itself? In this study, the researchers investigate this possibility.

**What Did the Researchers Do and Find?:**

The researchers collected synovial tissue from 55 patients with rheumatoid arthritis and used two approaches, called immunohistochemistry and real-time PCR, to investigate whether FDC-containing structures in synovium expressed an enzyme called activation-induced cytidine deaminase (AID), which is needed for both somatic hypermutation and class-switch recombination. All the FDC-containing structures that the researchers found in their samples expressed AID. Furthermore, these AID-containing structures were surrounded by mature B cells making ACPAs. To test whether these B cells were derived from AID-expressing cells resident in the synovium rather than ACPA-expressing immune system cells coming into the synovium from elsewhere in the body, the researchers transplanted synovium from patients with rheumatoid arthritis under the skin of a special sort of mouse that largely lacks its own immune system. Four weeks later, the researchers found that the transplanted human lymphoid tissue was still making AID, that the level of AID expression correlated with the amount of human ACPA in the blood of the mice, and that the B cells in the transplant were proliferating.

**What Do These Findings Mean?:**

These findings show that the ectopic lymphoid structures present in the synovium of some patients with rheumatoid arthritis are functional and are able to make ACPA. Because ACPA may be responsible for joint damage, the survival of these structures could, therefore, be involved in the development and progression of rheumatoid arthritis. More experiments are needed to confirm this idea, but these findings may explain why drugs that effectively clear B cells from the bloodstream do not always produce a marked clinical improvement in rheumatoid arthritis. Finally, they suggest that AID might provide a new target for the development of drugs to treat rheumatoid arthritis.

**Additional Information.:**

Please access these Web sites via the online version of this summary at http://dx.doi.org/10.1371/journal.pmed.0060001.

- This study is further discussed in a *PLoS Medicine* Perspective by Rene Toes and Tom Huizinga
- The MedlinePlus Encyclopedia has a page on rheumatoid arthritis (in English and Spanish). MedlinePlus provides links to other information on rheumatoid arthritis (in English and Spanish)
- The UK National Health Service Choices information service has detailed information on rheumatoid arthritis
- The US National Institute of Arthritis and Musculoskeletal and Skin Diseases provides Fast Facts, an easy to read publication for the public, and a more detailed Handbook on rheumatoid arthritis
- The US Centers for Disease Control and Prevention has an overview on rheumatoid arthritis that includes statistics about this disease and its impact on daily life

## Introduction

Rheumatoid arthritis (RA) is a chronic inflammatory erosive polyarthritis with an associated significant morbidity and mortality [1]. One of the hallmarks of the disease is the presence of circulating autoantibodies, such as rheumatoid factor (RF) [2] and anti-citrullinated protein/peptide antibodies (ACPA) [3–8], that have prompted the notion of an autoimmune pathogenesis. The presence of such antibodies, particularly ACPA, has been shown to be a poor prognostic factor linked with a higher erosive burden [9,10], while ACPA titres have been reported to fall in line with clinical response to biological therapies [11]. Additional support for a pathogenic role of ACPA antibodies comes from recent work in a mouse model of RA that indicated a direct role for ACPA antibodies in tissue destruction [12].

Physiologically, antigen-driven antibody responses take place within germinal centres (GCs) of secondary lymphoid organs where the processes of somatic hypermutation (SHM) and class switch recombination (CSR) of the Ig genes in GC-B cells occur, leading to affinity maturation and differentiation to memory B and plasma cells [13,14]. However, recent evidence suggests that CSR and low-level SHM can also be sustained at extrafollicular sites in the spleen [15] and the gut [16]. Both CSR and SHM are initiated by and critically dependent upon the expression of the enzyme activation-induced cytidine deaminase (AID) [17]. Although the exact mechanism of action of AID is currently unclear [18], CSR occurs via excision of switch circles following the introduction of double strand breaks in the Ig switch regions of DNA, with the subsequent substitution of IgM and IgD with IgG, IgE, or IgA [19], and hence production of antibodies with specific effector functions. AID also initiates SHM by introducing point mutations in the variable genes encoding the antigen-binding region of Igs [20], and affinity maturation then occurs through cycles of antigen-dependent selection in GC. Accordingly, AID expression in secondary lymphoid organs has been demonstrated to be restricted to GC-B cells actively undergoing CSR/SHM and to the recently described population of interfollicular (IF) large B cells [21–23]. Therefore, delineation of its expression within tissue allows identification of whether and where B cells activate the molecular machinery responsible for the production of affinity-matured antibodies.

The presence of ectopic lymphoid structures resembling GCs and characterized by follicular dendritic cell (FDC) networks is a common finding in chronically inflamed tissues in several autoimmune diseases [24] including RA. GC-like structures have been reported to occur in the synovial membrane in approximately 25% of patients with RA [25]. We and others have shown that alongside the frequent recognition of disorganized cell clusters in the synovium, some of the sublining lymphoid aggregates can recapitulate qualitative features of secondary lymphoid organs, such as FDC networks, the in situ production of homeostatic chemokines, as well as the acquisition of variable degrees of T/B cell segregation or high endothelial venule development [26–29]. However, whether ectopic lymphoid structures in RA can be functional and directly implicated in promoting the production of disease-specific and potentially pathogenic autoantibodies such as ACPA, or whether the synovium acts as a “reservoir” for long-lived plasma cells producing ACPA is at present unclear [30]. Circumstantial evidence, such as the presence of clonally mutated B cells from GC-like structures microdissected from RA tissues [31,32], suggests that an antigen-driven B cell response is taking place in the RA synovium. Likewise, the detection of ACPA in the synovial fluid of patients with RA [33,34], the demonstration that ACPA are concentrated at this site [35], and the detection of ACPA in the sera of SCID mice xenotransplanted with human synovium [36], support the possibility that ACPA might be directly generated in the synovium. These data, however, cannot rule out the possibility that already mutated, autoreactive B cells are preferentially recruited to the inflamed synovial tissue. In addition, lymphoid aggregates can also occur in the synovium of patients with other rheumatological conditions currently not associated with autoantibodies of known specificity[30], and further no correlation has been found between their presence and serum/synovial fluid ACPA [30,37]. These concepts have been taken as an indication that these structures may not be functional and that B cell autoimmunity in RA synovitis may be independent of ectopic lymphoneogenesis [30,37,38].

Thus, the purpose of this study was to investigate whether ectopic lymphoid structures in RA synovium are functional by determining whether they: (i) express AID, the enzyme required for SHM and CSR of Ig genes; (ii) support ongoing CSR and the production of high-affinity ACPA; and (iii) remain functional and promote B cell survival, proliferation, and autoantibody production in an RA/SCID chimera model devoid of any new immune cell influx into the synovium and where the ongoing immunological activity of secondary lymphoid organs is excluded.

## Materials and Methods

### Patients and Samples

Following written informed consent, synovial tissue was collected from 55 patients with RA fulfilling the revised 1987 ACR criteria for RA [39]. Tissues were collected consecutively within each group studied, which included arthroplastic joint surgery (40) and ultrasound-guided synovial biopsy (15). The majority of the patients were on methotrexate, and none were on anti-TNF therapy at the time of surgery. Demographic data are given for the study population in Table 1.

**Table 1:**
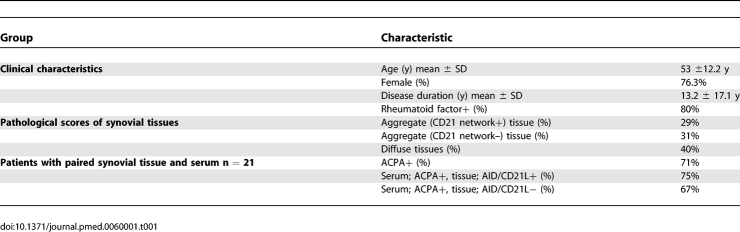
Clinical and Histological Characteristics of RA Patients (*n* = 55)

From 24 of the 55 patients who underwent joint replacement surgery between 1999 and 2005, only formaldehyde fixed, paraffin-embedded tissue was available and therefore was exclusively used for histological analysis. From an additional 25 patients (of whom ten underwent arthroplastic surgery and 15 underwent ultrasound-guided biopsy), enrolled between 2005 and 2008, paired paraffin and RNA samples were available and were used for histological analysis and QT-PCR. Finally, from six patients with RA undergoing joint replacement surgery, synovial tissue was obtained and stored in liquid nitrogen until use for transplantation into SCID mice.

Procedures were approved by the hospital Ethics Committee (REC/98/11/27 Guys and St. Thomas' NHS Trust and REC 05/Q0703/198 Barts and the London NHS Trust) and performed after written informed consent.

### Immunohistochemistry

A list of primary and secondary antibodies (Abs) used is reported in Table 2.

**Table 2:**
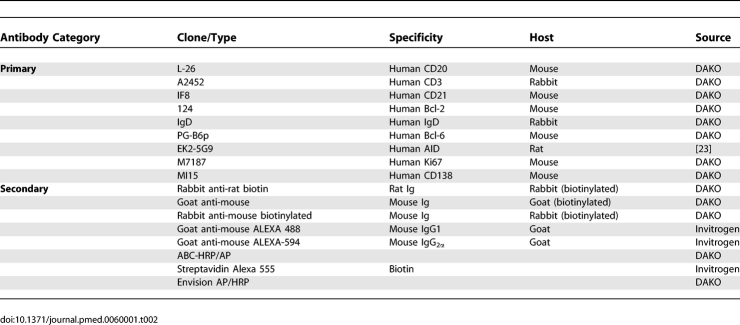
Primary and Secondary Antibodies Used for Immunohistochemistry

### Histological Grading of Tissues

The histological grading of tissues and degree of lymphoid organization was assessed by immunohistochemical staining of sequentially cut sections of RA synovial tissue, as previously reported [27]. Briefly, paraffin-embedded 5 μm sections underwent routine staining with haematoxylin and eosin in order to define the predominant histological pattern of RA synovitis as either diffuse or aggregate [25]. The number of lymphocytic aggregates was counted in each section and graded according to a modified, previously published grading system [27,40] with grade 1 (G1) aggregates displaying a radial cell number between 2 and 5 cells, grade 2 (G2) between 6 and 10 cells and grade 3 (G3) greater than 10 cells.

Staining for CD3 and CD20 (using antibody dilutions of 1:50 and 1:20 respectively), following antigen retrieval with Target retrieving solution (DAKO), was used to analyse T/B cell segregation as previously reported [41]. In addition, following proteinase K digestion (DAKO), single staining with CD21 at a dilution of 1:20 was performed to identify FDC networks characterising GC-like structures [41].

Based on the results of the CD21 staining and the presence of G2/G3 aggregates, samples were qualitatively classified as either diffuse, aggregate/CD21−, or aggregate/CD21+ as previously described [26]. In order to minimize bias due to cutting level, all blocks of tissue were cut and stained at three cutting levels 50 μm apart.

### Relationship between Histological Grade of Tissue, Degree of Inflammatory Infiltrate Organization, and AID Expression within Tissues

Staining for AID was performed, as previously reported [42], on sequential sections of the RA synovial tissue in order to correlate AID expression with the histological grading and degree of lymphoid organization. Briefly, after antigen unmasking using Target retrieval solution (pH 6, DAKO) and incubation with 20% rabbit serum (DAKO), incubation with the EK2-5G9 primary Ab was carried out at a dilution of 1:20 for 1 h at room temperature. This step, in turn, was followed by a 1 h incubation with the biotinylated rabbit anti-rat Ig secondary Ab. The HRP–streptavidin-biotin complex (DAKO) was then added to the section and incubated for 30 min. The colour reaction was developed with diaminobenzidine (DAKO).

### Relationship between AID and CD21L Expression, as Determined by QT-PCR and Histological Characterization of Synovial Tissues

Synovial samples were obtained from an additional 25 patients with RA (the same extra 25 mentioned above). Each specimen was divided into 2 parts; one was formalin fixed and paraffin embedded for immunohistology and the second was stored in a 10:1 v:v of RNA-later (Ambion) at −80 °C for RNA extraction and QT-PCR analysis. Histological characterization of the RA tissue was carried out as described above. Total RNA was extracted from the remaining portion of synovial tissue, using the RNeasy Mini Kit (Qiagen), with on column DNase I digestion to avoid genomic DNA contamination. cDNA was generated from 1 μg of RNA using the Thermoscript RT-PCR System for First-Strand cDNA Synthesis (Invitrogen). QT-PCR was performed to detect mRNA expression levels of AID and CD21L together with CXCL13, LTβ, BAFF and APRIL using specific primers and probes (see Table 3). The RT-PCR was run in triplicate with an equal loading of 20 ng of cDNA/well. Results were analysed after 40 cycles of amplification using the ABI PRISM 7900HT Sequence Detection System Version 2.1. Relative quantification was measured using the Comparative C_T_ (Threshold Cycle) Method. cDNA from peripheral blood mononuclear cells sorted for CD3 by fluorescence activated cell sorting (FACS) were used as a negative control for AID and CD21L, whereas cDNA from lymph node was used as a positive control.

**Table 3:**
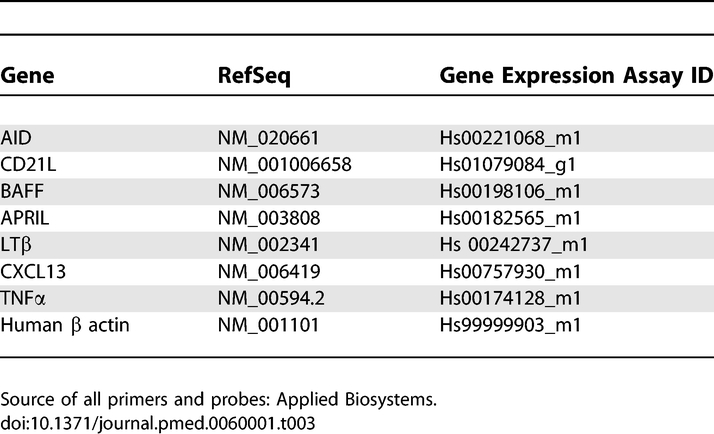
Genes, Specific Primers, and Probes used for QT-PCR

### Detection and Characterization of ACPA-Producing Cells within Rheumatoid Synovial Tissue

Citrullinated Fb (CFb) was generated as previously described with minor modifications [43,44]. Briefly, plasminogen-depleted human Fb (Calbiochem: EMD BioSciences) was incubated at 0.86 mg/ml with 10 U/ml of rabbit skeletal muscle peptidyl arginine deiminase (Sigma-Aldrich) in 0.1 M Tris-HCl (pH 7.4), 10 mM CaCl_2_, and 5 mM DTT for 2 h at 50 °C. The enzyme was inactivated by adding 2% SDS and heating at 100 °C for 3 min and removed by serial spinning and washing with 0.01 M sterile bicarbonate buffer in a 100 kDa Amicon filter device (Millipore). CFb was then biotinylated using NHS-LC biotin (Pierce Biotechnology). In addition, an aliquot of unmodified Fb of the same concentration as the CFb was biotinylated and used as a negative control for all staining experiments.

To confirm effective citrullination and the specificity of CFb for ACPA Abs, two aliquots of CFb and uncitrullinated Fb were first separated by SDS-PAGE on 7.5% polyacrylamide gels. Proteins were then electrotransferred onto Hybond-C extra reinforced nitrocellulose membranes (Amersham). Membrane strips were probed with human sera (1:100) pooled from three ACPA+ patients with RA and three ACPA– patients with RA. A goat anti-human IgG peroxidase (Sigma) was used for detection of the primary Ab. Peroxidase activity was visualized using ECL Western blotting reagents (Amersham) following the manufacturer's instructions.

To detect and characterise ACPA-producing cells in the RA synovium, sequentially cut 5μm paraffin and/or frozen sections of five RA synovial samples representative of different degrees of histological organization (three aggregate/CD21+, one aggregate/CD21−, one diffuse) were chosen for analysis. ACPA-producing cells and B cell follicles were identified using double immunofluorescence with biotinylated CFb followed by incubation with streptavidin ALEXA-555 and CD20 followed by rabbit anti-mouse ALEXA-488 with subsequent DAPI counterstaining [43]. AID+ FDC networks were identified on sequential sections by staining for CD21 using conventional IHC as described above and immunofluorescent staining for AID using streptavidin-ALEXA-555 following incubation with biotinylated rabbit anti-rat secondary Ab. Plasma cells producing ACPA were identified by performing double immunofluorescence for CD138 followed by goat anti-mouse ALEXA-488 and biotinylated CFb as described above. All sections were visualised using an Olympus BX60 microscope and epifluorescence.

### Tissue Transplantation

A total of 56 samples of human synovium from six patients with RA undergoing arthroplasty (classified prior to transplantation histologically as two diffuse, two aggregate/CD21−, and two aggregate/CD21+) were transplanted subcutaneously into Beige SCID-17 mice as previously described [45,46]. Each mouse was double transplanted with tissue obtained from adjacent pieces of synovium from the same patient, thus minimizing variability of transplants within each mouse. Thirty-four mice were double transplanted. From the total of 68 transplants, 12 could not be used for the analysis due to either resorption of the transplant or poor RNA yield. (Such a rate of graft loss is not uncommon in our experience, thus in order to keep animal numbers to a minimal level, double transplantation is normal practice in our laboratory.) In this model, full engraftment of human tissue is reached at 7 d, and significant levels of ACPA antibodies in mouse sera have been described from a similar time point and up to 84 d [36]. Four weeks post-transplantation we determined: (i) the histomorphology of the transplanted synovium with regard to the presence of CD21+ aggregates; (ii) the mRNA expression levels of AID, CD21L, and genes involved in ectopic lymphoneogenesis; (iii) the presence of ongoing CSR by assaying for the presence of Iγ-Cμ circular transcripts (molecular by products of IgM to IgG class switching); and (iv) the serum levels of human ACPA in the mouse circulation.

Animals were humanely killed at 4 wk and the grafts divided into two parts; one part was paraffin embedded for later histological examination and one part was stored in 10:1 v:v of RNA-later (Ambion) at −80 °C for QT-PCR. Grafts were examined macroscopically and histologically to confirm viability. In addition, transplanted synovial tissues were characterised by staining for CD21 and AID to identify persisting AID+ FDC networks. Proliferating B cells were identified by performing double immunofluorescence for Ki67 followed by goat anti-mouse IgG_1_ ALEXA-488 and CD20 followed by goat anti-mouse IgG_2a_ ALEXA-594.

Finally, mice underwent a terminal bleed, and serum was collected and stored at −20 °C for subsequent ACPA analysis.

### Gene Expression Analysis by QT-PCR and Detection of Circular Transcripts in Transplanted RA Synovial Tissues

RNA was extracted from each transplant, and cDNA was generated as described above. QT-PCR was performed to measure mRNA expression levels of AID, CD21L, APRIL, BAFF, TNFα, LTβ, and CXCL13 (see Table 3).

Detection of Iγ-Cμ circular transcripts was performed on the same cDNA. Circular transcripts were detected following 30 cycles of RT-PCR amplification using the reverse primer Cμ (5′-GTTGCCGTTGGGGTGCTGGAC-3′) together with the forward primer Iγ (5′-GGGCTTCCAAGCCAACAGGGCAGGACA-3′) recognizing both Iγ1 and Iγ2 (the RT-PCR product was 557 bp) as previously reported [47]. Before each RT-PCR, cDNA were denatured for 5 min at 94 °C. The PCR conditions were as follows: denaturation for 1 min at 94 °C, annealing for 1 min at 60 °C, and extension for 1 min at 72 °C.

### Detection of Human IgG ACPA in Human and Mouse Sera

ACPA were detected in both mouse and human sera using a commercially available anti-cyclic-citrullinated antibody (anti-CCP2) ELISA kit (Axis Shield), following the manufacturer's instructions. A positive result was taken as > 5 U/l.

### Statistical Analysis

Differences in quantitative variables were analysed by the Mann Whitney U test when comparing two groups, and by the Kruskal-Wallis with Dunn's post test when comparing multiple groups. χ^2^ test with Yates' correction when required or Fisher's exact test when appropriate were used to evaluate associations of qualitative variables in the different groups. Spearman's rank correlation was performed to correlate expression levels of AID and CD21L mRNA in RA synovial grafts. All the statistical analyses were performed using GraphPad Prism version 3.03 for Windows (GraphPad Software). A *p*-value of < 0.05 was considered statistically significant.

## Results

### In the Rheumatoid Synovial Membrane AID Is Expressed Only in Association with FDCs

In order to investigate whether AID was expressed within the rheumatoid synovial membrane and its potential association with specific patterns of rheumatoid synovitis, we first performed an IHC analysis on sequential sections of synovial tissue in 24 patients with RA. We analysed AID expression in relation to the degree of lymphoid organization of the inflammatory infiltrate, including T/B cell segregation and FDC network formation.

**Figure 1:**
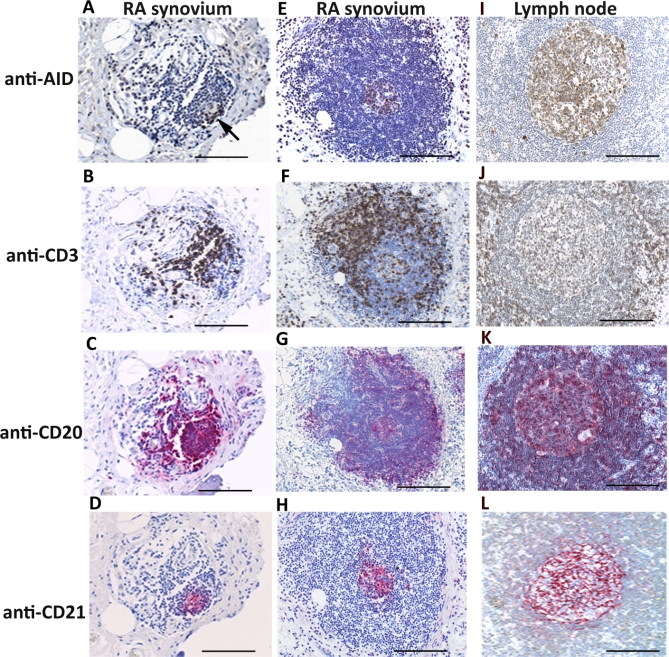
AID Expression within the Rheumatoid Synovial Membrane Is Restricted to Lymphoid Aggregates with FDC Networks A representative example of sequential paraffin-embedded sections of LN and RA synovial membrane stained for AID (brown, A, E, I), T cells (CD3, brown, B, F, J), B cells (CD20, red, C,G, K) and follicular DCs (CD21, red, D,H, L). The presence of FDC networks was invariably associated with the expression of AID even in FDC+ grade 3 aggregates (arrow, A), without morphologically detectable dark and light zones within the B cell rich area (A−D). In more organised grade 3 aggregates (E−H), expression was more prominent and closely resembled that seen in secondary lymphoid organs, such as the LN (I−L) (original magnification was 20×). Scale bars 200 μm.

As shown in a representative example in Figure 1, AID expression in RA synovium was invariably associated with the presence of FDCs (Figure 1A–1H). This was the case even in CD21+ G3 aggregates that lacked uniform T/B cell segregation and distinct dark –light zones within the B cell rich area [32] (Figure 1A–1D). The pattern of AID staining within compartmentalized CD21+ G3 aggregates (Figure 1E–1H) was highly reminiscent of the pattern seen in secondary lymphoid organs, such as lymph node, with strong AID expression within GC B cells (Figure 1I–1L). CD21+ FDC networks were observed in seven out of 24 patients (29%), with 100% of G3 aggregates displaying CD21+ FDC networks also expressing AID. Conversely, AID and FDC networks were not detectable either in smaller G1 or G2 aggregates or in diffuse tissues.

### QT-PCR Evaluation Confirms the Exclusive Association between AID and FDCs

**Figure 2:**
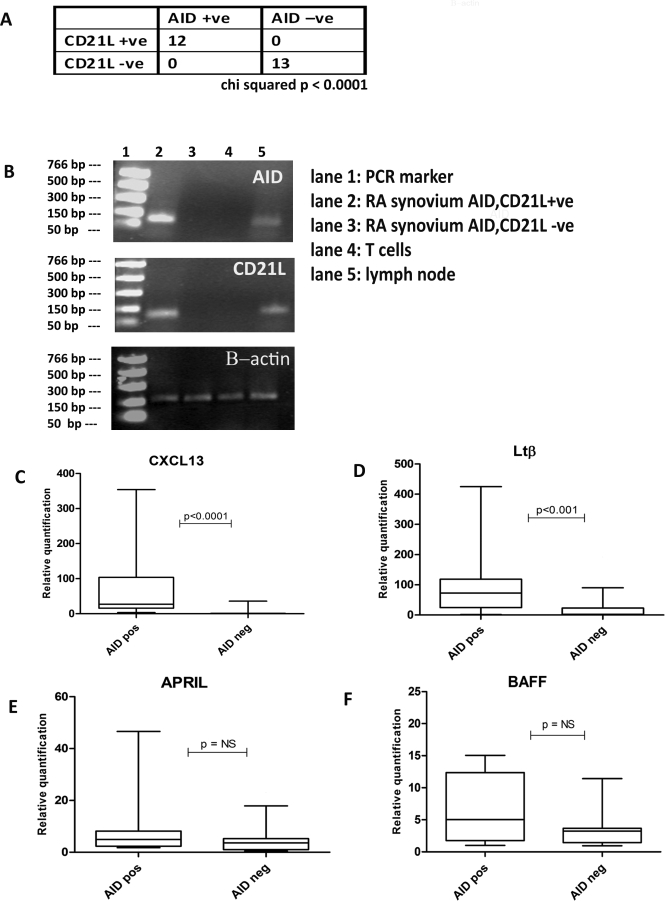
AID mRNA within Rheumatoid Synovial Tissue Is Expressed Exclusively in Association with CD21L-Isoform Transcripts QT-PCR was used to measure levels of transcripts of AID and CD21L in synovial samples from 25 patients with RA. Results were normalized for the endogenous control (human β-actin) and expressed as relative quantification. (A) The presence of AID is restricted to those tissues expressing CD21L. (B) Representative examples of the PCR products were run in a 1.8% agarose gel to ensure the presence of a single specific amplification product and confirm specficity of QT-PCR. Amplicons of the expected length (99 bp for AID and CD21L and 171 bp for β-actin) were observed in samples that gave positive signals at QT-PCR (lane 2), while no bands were detected in samples negative for AID/CD21L by QT-PCR (lane 3). No AID/CD21L amplification was detectable in negative controls (CD3-sorted cells, lane 4), while AID and CD21L were both detectable in control human lymph node (lane 5). (C–F) CXCL13, LTβ, BAFF, and APRIL transcript analysis by QT-PCR. AID expression within synovial tissue was associated with the up-regulation of both CXCL13 and LTβ but no significant difference was seen in the levels of APRIL and BAFF. The lower and upper margins of the box represent the 25th and 75th percentiles, with the extended arms representing the 10th and 90th percentiles, respectively. The median is shown as a horizontal line within the box. *p*-Values were calculated using the Mann-Whitney U test.

In order to confirm whether AID expression was also exclusively associated with FDC networks at the mRNA level, we analysed synovial samples from an additional cohort of 25 patients with RA in which paired RA specimens for IHC and QT-PCR were available. In this cohort, we investigated the expression of CD21L, a marker exclusively expressed by FDCs [48], and AID transcripts. Indeed, AID mRNA expression was detected in all 12 of the CD21L+ RA samples but in none of the 13 CD21L− RA samples analysed (Figure 2A, *p* < 0.0002). Amplicons of the expected length (99 bp for AID and CD21L, and 171bp for β-actin, Figure 2B) were seen in samples positive for AID and CD21L by QT-PCR (lane 2) and human lymph node (lane 5), but not in RA samples negative for AID and CD21L (lane 3) or in the negative control (lane 4, cDNA from CD3 sorted cells).

In the same cohort of 25 patients we compared the expression of AID and CD21L mRNA by QT-PCR with histological characterization of the same piece of synovial tissue. We identified by IHC an aggregate pattern AID+/CD21+ in seven patients (28%), aggregate pattern AID−/CD21− in seven (28%) patients, and diffuse infiltrate negative for both AID and CD21 in 11 patients (44%). On the other hand, when the same samples were analysed by QT-PCR, we demonstrated a higher prevalence of RA synovial tissues expressing AID and CD21L mRNA (12/25, 48%). Notably, the increased proportion of RA synovial tissues expressing AID and CD21L mRNA was exclusively characterised by an aggregate and not a diffuse pattern, confirming that lymphoid structures are required for the expression of both AID and CD21L mRNA in the RA synovial membrane.

### Expression of AID Is Associated with the Up-regulation of CXCL13 and LTβ within the Rheumatoid Synovial Membrane

As synovial tissues containing sites of ectopic lymphoneogenesis have been demonstrated to be closely associated with increased expression levels of CXCL13 and LTβ mRNA [26], we then sought to determine whether the expression levels of these and other key factors involved in lymphoneogenesis were also significantly associated with the up-regulation of AID and CD21L mRNA. As shown in Figure 2, AID mRNA+ tissues displayed significantly higher levels of CXCL13 and LTβ mRNA (Figure 2C and 2D) but not of the B cell survival factor BAFF (Figure 2F). Conversely, although APRIL mRNA showed an increase in AID+ synovial tissue, these differences did not reach statistical significance (Figure 2E).

### AID Identifies Interfollicular Large B Cells within the Rheumatoid Synovial Membrane in Association with FDC-Positive Aggregates

**Figure 3:**
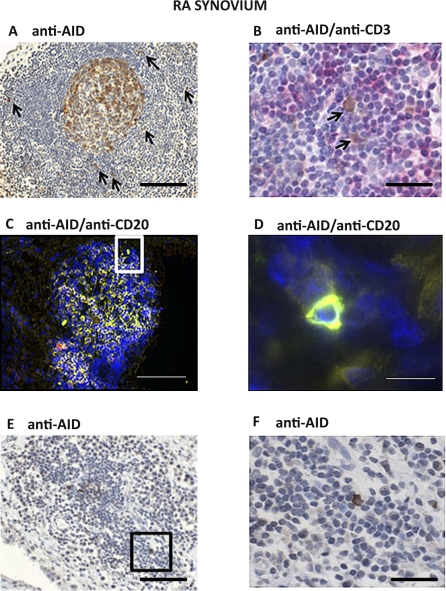
AID Expression within the Rheumatoid Synovial Membrane Identifies Interfollicular Large B Cells (A) Paraffin-embedded sections from RA patients were stained for AID (20× magnification). AID+ cells with a large cytoplasm and sometimes dendritic-like morphology (arrows) were frequently and exclusively seen in RA synovial tissue characterized by the presence of large aggregates predominantly in close proximity to CD21+ FDC networks. (B) Paraffin sections were double stained for AID (brown) and CD3 (red) (60× magnification), demonstrating the close relationship between AID positive cells (arrows) and T cells in the peripheral T cell areas of the lymphoid aggregates. (C) Merged double staining for AID (red) and CD20 (green) on frozen RA sections confirmed that AID+ cells were of B cell origin (double-stained cells are identified in yellow). (D) Higher magnification of an example of an AID+/CD20+ cell (60× magnification of [C]). (E and F) Scattered AID+ cells (E) with the appearance of IF B cells (F) were occasionally found away from the central focus of the aggregate. Scale bars: 200 μm (A, C, E), 50 μm (B, F), 15 μm (D).

In addition to AID+ B cells within FDC+ networks, a second set of cells within the synovial membrane expressed AID outside this compartment. These cells were characterised by a large cytoplasm, often with a dendritic morphology, and were localized in close association with neighbouring T cells exclusively in CD21+ aggregates (Figure 3A and 3B). Using double immunofluorescence for AID and CD20 (Figure 3C and 3D), we confirmed that these AID+ cells were of B cell origin. The morphological appearance, tissue distribution, and predominant cytoplasmic AID expression in these cells (Figure 3E and 3F) closely resembles the population of IF large B cells recently described in lymph nodes [22] where AID+ IF large B cells are mainly localised within the T cell area surrounding the follicles [21,23].

### AID-Positive Follicles Are Surrounded by Plasma Cells Producing ACPA

We next investigated whether AID+/FDC+ GC-like structures within the rheumatoid synovial membrane were associated with the in situ production of ACPA.

**Figure 4:**
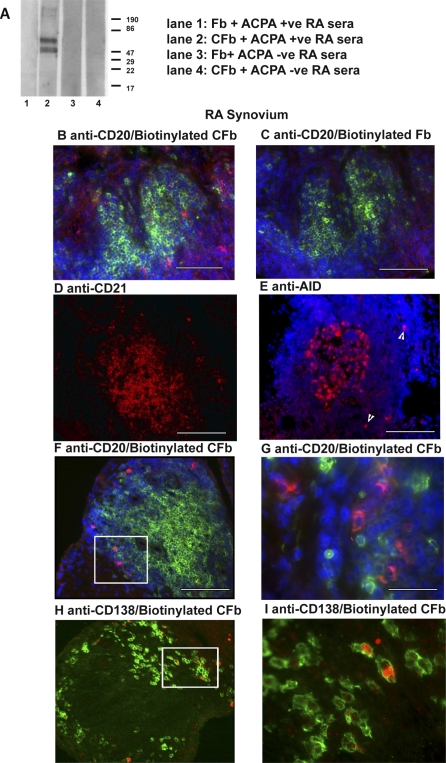
AID+ Aggregates Are Surrounded by Plasma Cells Producing ACPA within Rheumatoid Synovium (A) Immunoblot analysis of reactivity of pooled ACPA+ and RA− sera to citrullinated fibrinogen (CFb) (lanes 2 and 4) and control non-citrullinated fibrinogen (Fb) (lanes 1 and 3). Two protein bands between 60–80 kDa in size, corresponding to the α- and β-chains of CFb, are recognized by the ACPA+ RA sera (lane 2) but not control Fb (lanes 1). ACPA− sera (lanes 3 and 4) show no immunoreactivity toward either citrullinated or control Fb. (B–I) Double or single immunofluorescence analysis showing phenotypic characterization and immunoreactivity towards citrullinated proteins within RA synovial tissue. Sequential sections of RA synovial tissue were double stained with anti-CD20 to detect B-cells (green) together with either biotinylated CFb (B, red) or control biotinylated Fb (C). Sections were counterstained with DAPI. Positive staining is only seen in the sections incubated with biotinylated CFb, confirming the presence of anti-citrullinated protein immunoreactivity. (D–I) Sequential sections of rheumatoid synovium stained with anti-CD21 to detect FDC (D), anti-AID (E), double stained with anti-CD20 (green) and biotinylated CFb (red) (F, G) and double stained with anti-CD138 (green) to detect plasma cells and biotinylated CFb (red) (H, I). CD21 was visualized with vector red. AID, CD20, CD138, and biotinylated CFb were incubated with secondary Abs/streptavidin conjugated to ALEXA fluorochromes. AID and CD20 were counterstained with DAPI. ACPA Ab-producing cells were seen scattered around FDC and AID+ aggregates (F). ACPA+ cells were consistently CD20− (G) (60× magnification of F) and CD138+ (H) (60× magnification of G). Original magnification was 20× (B–F, H). Scale bars: 200 μm (D–F, H), 50 μm (B, C) 20 μm (G, I).

Biotinylated citrullinated fibrinogen (CFb) was produced as previously reported [43] and tested for specificity using pooled sera from three ACPA+ and three ACPA− patients with RA. As expected, ACPA+ sera showed reactivity to CFb but not to unmodified Fb (Figure 4A, lanes 1 and 2), whilst no reactivity was observed with ACPA− sera (Figure 4A, lanes 3 and 4). Biotinylated CFb and biotinylated Fb were then used to detect in situ production of ACPA in sections double stained for CD20 (Figure 4B and 4C). Numerous positive cells were detected with CFb (Figure 4B) but not when using Fb as a negative control (Figure 4C) in ACPA+ patients. Conversely no staining was seen around germinal centres from a sample from an ACPA− patient (data not shown).

ACPA+ cells were localised around AID+/CD21+ aggregates (Figure 4D, 4E and 4F), were CD20− (Figure 4F and 4G) and CD138+ (Figure 4H and 4I), confirming their plasma cell origin. These results suggest that AID+ GC-like structures within rheumatoid synovial tissue can support the local differentiation of ACPA-producing plasma cells.

### AID Expression Is Maintained within RA Synovial Grafts in the HuRA-SCID Mouse Chimera Model, Sustains Ongoing CSR and Supports the Production of ACPA

**Figure 5:**
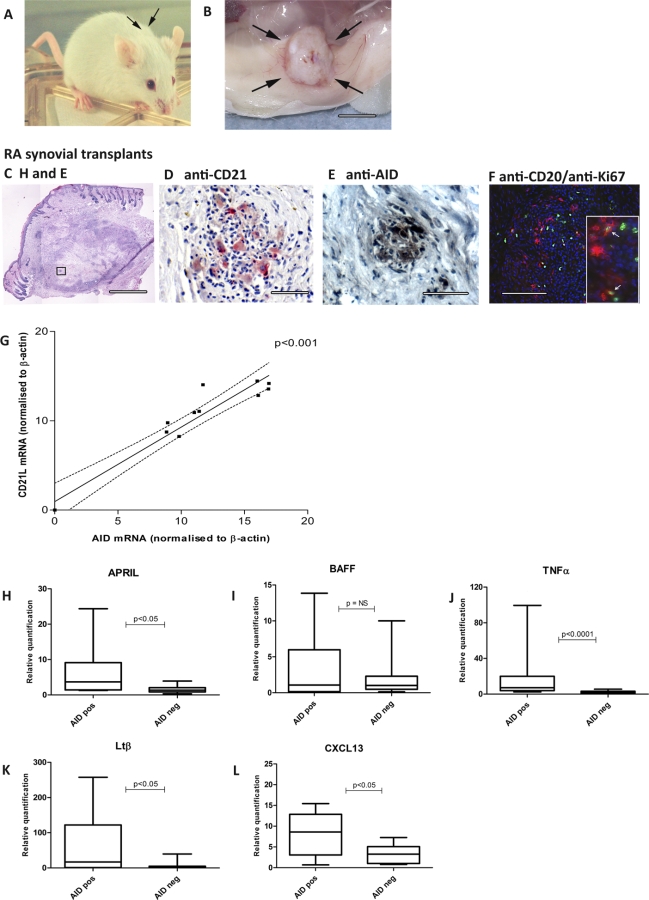
Rheumatoid Synovial Grafts in SCID Mice Maintain AID Expression and the Up-regulation of Genes Determining Lymphoneogenesis (A) Rheumatoid synovial tissue (*n* = 56) was transplanted into SCID mice (arrows depict site of transplants). (B) Transplanted RA synovial tissue in SCID mouse. (C–F) Montage image of a paraffin embedded haematoxylin and eosin stained section of transplanted tissue depicting the whole graft (original magnification 4×, area outlined in black equates to aggregate seen at higher magnification in [D] and [E]). Sequential sections of transplants stained using conventional immunohistochemistry for CD21 (D), AID (E), and double immunofluorescence for Ki67 (green) and CD20 (red) (F) (inset at 40× magnification, with arrowheads indicating the cells staining positively for Ki67 and CD20) demonstrated persistent FDCs, associated expression of AID (original magnification 40×) and continued proliferation of B cells. Four weeks post-transplantation, total RNA from the grafts was reverse transcribed and used for QT-PCR to determine the mRNA expression levels of AID and CD21L and a number of genes known to correlate with lymphoid tissue organization. (G) A significant correlation between levels of transcripts for AID and CD21L was found and AID was detected only in the presence of CD21L. (H–L) Tissues were stratified according to the presence or absence of AID as determined by QT-PCR, and analysed for the expression of the selected cytokines/chemokines genes between the groups. A significant difference is seen in levels of APRIL (H), CXCL13 (L), TNFα (J), and LTβ (K). Significant *p*-values are depicted, Mann-Whitney U test. No significant difference is seen between levels of BAFF (I). The lower and upper margins of the box represent the 25th and 75th percentiles, with the extended arms representing the 10th and 90th percentiles, respectively. The median is shown as a horizontal line within the box. Scale bars: 6 mm (B) 2 mm (C), 50 μm (D, E, F).

The histomorphological analysis described above suggested that ectopic AID+/CD21+ aggregates are capable of sustaining in situ ACPA production in the RA synovium. We next examined a) the direct functional relationship between the synovial expression of AID and CD21L and the production of ACPA and b) whether ectopic lymphoid aggregates within RA synovium maintain functionality independently from incoming immune cells from the periphery. To this end, we generated Hu-RA SCID mice chimeras by implanting fragments of RA synovial tissue into these animals (Figure 5A and 5B), and analysed the results at 4 wk.

#### (i) AID/CD21L expression and B cell proliferation are maintained within RA synovial grafts.

Histomorphology and immunohistological analysis of paraffin-embedded sections of transplanted RA synovium demonstrated viable grafts with residual inflammatory infiltrates (Figure 5C), often characterized by persistent FDC networks and associated AID expression (Figure 5D and 5E). Importantly, AID was detected only in the presence of CD21L in synovial grafts and AID mRNA expression levels closely correlated (r=0.88) with those of CD21L (Figure 5G). In addition to the expression of AID, further evidence of functional activation of B cells within synovial grafts was determined by performing double staining for the B cell marker CD20 and Ki67, a marker of cell proliferation. Double stained, proliferating B cells were identified within aggregates within grafts at 2 wk (Figure 5F), confirming that B cells within synovial grafts are able to both survive and proliferate in this micro environment, and hence, retain the capacity to function as GC B cells.

#### (ii) AID and CD21L expression in RA synovial grafts is associated with the up-regulation of genes regulating ectopic lymphoneogenesis.

To evaluate whether the sustained expression of AID and CD21L mRNA in RA synovial grafts was associated with dynamic gene expression regulating ectopic lymphoneogenesis, we analysed mRNA expression levels of CXCL13, LTβ, TNFα, APRIL, and BAFF. As shown in Figure 5H-5K, expression levels of the TNF family members LTβ, TNFα, and APRIL, as well as the lymphoid chemokine CXCL13 were significantly higher in AID+ RA grafts as compared to AID− grafts. Conversely, although BAFF was abundantly expressed in all RA synovial grafts, no significant differences between AID+ and AID− grafts were found. These results are in keeping with previous reports [49], showing no difference in the expression patterns of BAFF between CD21+ and CD21− synovial tissues, but an up-regulation of APRIL mRNA in GC+ synovitis, and indicate that ectopic lymphoneogenesis in RA synovium develops and is maintained in the presence of high expression levels of these factors.

#### (iii) AID and CD21L expression in RA synovial grafts is associated with ongoing CSR and in situ production of ACPA antibodies.

**Figure 6:**
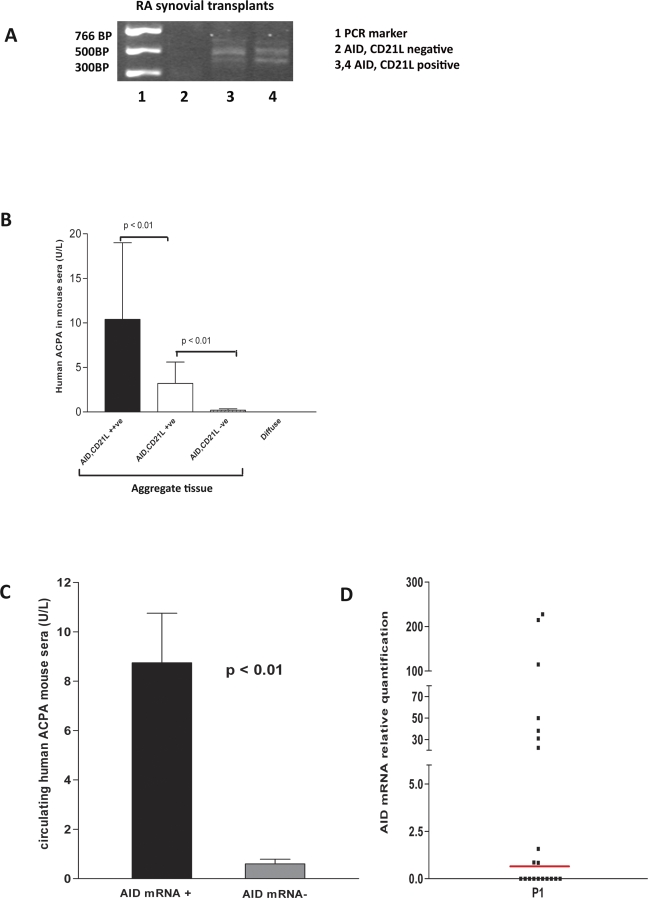
ACPA Production in the HuRA-SCID Mouse Is Associated with Functional AID Expression within Synovial Grafts Specimens of synovium from six patients with RA were transplanted into 34 SCID mice. Four weeks post-transplantation mice were killed and all sera tested for the presence of ACPA, while mRNA from the grafts (*n* = 56) was reverse transcribed and used for QT-PCR to determine the number of transcripts for AID and CD21L, as well as for RT-PCR to determine the presence of circular transcripts. (A) A 1.8% agarose gel showing representative PCR products from RT-PCR performed for Iγ-Cμ circular transcripts (double bands representing alternatively spliced transcripts and confirming specificity [47]) indicating ongoing CSR in synovial grafts positive for AID/CD21L and producing ACPA (lane 3 and 4) but not in transplants negative for AID/CD21L (lane 2). (B) Synovial transplants from six RA patients were grouped according to histological classification, as aggregate versus diffuse, and activation-induced cytidine deaminase, CD21L mRNA expression as high (++), low (+), and negative (−). Significantly higher levels of anti-citrullinated protein/peptide antibodies were seen in those mice transplanted with synovium expressing high mRNA levels of AID/CD21L. Notably, ACPA were not seen in the absence of AID. (C and D) To establish the relationship within the same patient between levels of AID expression and ACPA production, synovial grafts from an illustrative patient (P1) were stratified according to the presence or absence of AID as determined by QT-PCR (D) (horizontal line indicates cut off for positivity). Circulating ACPA from synovial grafts were produced at a significantly higher level in AID+ compared to AID− grafts. Error bars indicate mean levels of ACPA + SD. *p*-Values were calculated using Kruskal-Wallis test (B) and Mann Whitney U test (C).

In order to confirm that AID expression in RA synovial grafts was functional, evidence of ongoing CSR was sought by analysing for the expression of circular transcripts. Circular transcripts are known to be specifically associated with AID expression and exclusively detectable in B cells undergoing CSR, but not by plasma cells, which have already undergone CSR [50]. By performing RT-PCR for Iγ-Cμ circular transcripts, which are transiently produced for around 48 h following class switching from IgM to IgG [50], we demonstrated (Figure 6A) their invariable detection in those RA transplants also expressing AID and CD21L mRNA, confirming the functionality of AID expression in synovial grafts.

#### (iv) Levels of human ACPA in the mouse circulation are associated with intra-graft expression of AID and CD21L mRNA.

Finally, we investigated whether there was a direct relationship between AID and CD21L mRNA expression within transplanted RA synovial grafts and the production of class-switched human IgG ACPA detectable in the mouse circulation. Strikingly, ACPA were only detected in the sera of mice transplanted with RA synovial tissues expressing AID and CD21L mRNA (Figure 6B). In addition, the highest levels of ACPA in the serum were detected in the RA transplants that were histologically characterized by an aggregate pattern and high levels of AID mRNA expression (Figure 6B). The levels of ACPA decreased in line with the decrease of AID mRNA expression, whilst no ACPA were detected in AID− grafts with a diffuse pattern (Figure 6B). To establish the relationship within the same patient between the variable levels of AID expression and ACPA production, multiple synovial grafts (*n* = 20) obtained from the same joint of the same patient with RA were stratified according to the presence or absence of AID as determined by QT-PCR (Figure 6D, P1). We demonstrated that circulating ACPA from synovial grafts were produced at a significantly higher level in AID+ grafts, while ACPA were negligible in the serum of animals transplanted with AID− grafts (Figure 6C). These data indicate that, despite the known variability between different synovial biopsies within the same patient, a functional relationship between AID expression and ACPA production is maintained.

## Discussion

In this report we provide strong evidence that lymphoid structures in the target organs of a human autoimmune disease are functional by demonstrating that within the synovial membrane of patients with RA, ectopic lymphoid aggregates characterized by FDC networks invariably express AID and are surrounded by ACPA producing plasma cells. In addition, using the human RA-SCID mouse chimera model, we demonstrate that transplanted RA synovial grafts containing ectopic lymphoid structures support B cell survival and proliferation, maintain AID expression, continuously promote ongoing CSR and produce human IgG ACPA. This phenomenon, characterized by the sustained high expression levels of genes regulating ectopic lymphoneogenesis and in the absence of new influx of immune cells, indicates that the establishment of follicular structures in the synovium can lead to self-perpetuating autoimmunity. The self-sustained survival of B cell niches, within rheumatoid synovium, may also have a critical impact on the capacity of B cell–depleting biologics to modulate chronic inflammation and autoimmunity.

The elucidation of the mechanisms coupling chronic inflammation and the production of disease specific autoantibodies within RA synovium is of pivotal importance because ACPA are highly disease specific markers of RA, are strongly associated with a more destructive arthritis and are independent predictors of a poorer prognosis [9,10]. However, despite recent evidence that ACPA can mediate tissue damage in animal models [12], confirmation of a direct pathogenic role for ACPA in patients with RA is currently lacking. The presence of ectopic GC-like structures, characterized by T/B cell aggregates and CD21+ FDC networks, in the synovium of a subset of patients with RA has long been known. These structures have been associated with the expression of cellular and molecular markers associated with GC responses in secondary lymphoid organs [26,27,51] and analysis of Ig sequences from microdissected GCs from synovial tissue has demonstrated hypermutated and clonally related sequences [31], suggesting that an antigen driven B cell response is ongoing within RA synovium. Nonetheless, direct evidence that ectopic lymphoid tissue in RA synovium contains the required molecular machinery to sustain ongoing CSR and affinity maturation of autoantibodies has so far been lacking. Recent evidence has demonstrated that the processes of SHM and CSR are critically dependent on the expression of the enzyme AID [17] and that AID expression is exclusively associated with B cells undergoing SHM and CSR [52]. Therefore delineating its expression pattern has allowed us to establish that B cells activate the molecular machinery responsible for production of affinity matured antibodies in follicular structures within the RA synovial membrane.

In this study we provide, to our knowledge, the first demonstration that AID is invariably expressed within rheumatoid synovial T/B cell aggregates containing CD21+ FDC networks, with a distribution closely recapitulating that seen in secondary lymphoid organs, providing direct evidence that ectopic GC-like structures represent a functional tertiary lymphoid organ capable of activating the molecular machinery necessary to sustain SHM and CSR within the synovial membrane. Accordingly, ongoing CSR, as demonstrated by the expression of circular transcripts, was invariably detectable within AID+/FDC+ RA synovial tissues. These results confirm and expand our recent observations in Sjögren's syndrome [42] in which FDCs appeared necessary for sustaining AID expression in the ectopic lymphoid tissue found in salivary glands, where an antigen-driven B cell response can occur [53]. This evidence, further supported by our data demonstrating the presence of AID transcripts only in the presence of CD21L mRNA, strongly supports the conclusion that within the rheumatoid synovial membrane, AID requires the presence of FDCs for expression. We also provide further histological evidence supporting both the functionality of these AID+ structures and their involvement in the production of ACPA by showing that these structures are surrounded by ACPA producing plasma cells.

In addition to the expression of AID by B cells within ectopic GC-like structures, we also report the novel observation that the expression of AID within synovial tissue identifies a newly characterised subset of B cells, defined as IF large B cells [21]. These cells have been shown to express AID outside of GCs in secondary lymphoid organs [21,23], while the presence of somatic mutations within their Ig genes [22] supports AID functionality. Importantly, among the RA samples that we analysed, AID+ IF large B cells were found exclusively in synovial aggregates containing ectopic GCs, raising the possibility that they may represent a population of post-GC B cells. In addition, they share many similarities with IF large B cells in secondary lymphoid organs, namely their localisation within the peripheral T cell area, a predominantly cytoplasmic AID staining pattern and a dendritic morphology [22]. We have recently reported their presence in ectopic GC-like structures in salivary glands of patients with Sjögren's syndrome [42], suggesting that these cells are associated with sites of ectopic lymphoneogenesis in the target organs of several autoimmune disorders. The role of IF large B cells is currently unknown, but their dendritic-like morphology and close association with T cells has led to speculation that they may play an important role as antigen presenting cells [22].

By evaluating the expression of CD21L and AID mRNA using QT-PCR, a sensitive and highly specific method, we were able to identify functional sites of ectopic lymphoneogenesis in about 50% of the RA synovial samples. This prevalence was significantly higher than that observed using histological analysis and semiquantitative PCR reported in approximately 25% [26], and is in line with a recent observation by other groups [54]. These additional (low) CD21L+ tissues were always of aggregate appearance, and were invariably accompanied by AID expression and ongoing CSR, strongly supporting the concept that such aggregate structures are also functional. These results demonstrate that the presence of lymphoid aggregates and ongoing GC reactions in RA synovium is a phenomenon considerably more common than has recently been proposed [30,37,38].

In this report, by using the HuRA-SCID mouse model we were able to provide direct evidence of functionality and to present a number of novel findings regarding the production of ACPA within the rheumatoid synovial membrane. First, as in the original RA synovial tissue, the expression of AID within the transplanted tissue was consistently associated with the presence and levels of CD21L. This, in turn, was invariably associated with ongoing CSR and the production of human IgG ACPA detectable in the mouse sera. In addition, levels of AID within the RA transplants directly correlated with the levels of ACPA in the mouse sera. Finally, even low-level expression of AID and CD21L within the transplants was still associated with significant ACPA detection in the sera, while ACPA were barely detectable in the absence of AID mRNA expression in the RA grafts. Overall, these results strongly support the notion that lymphoid structures expressing AID and CD21L can directly contribute to ACPA production within the rheumatoid synovial membrane. Indeed, as further evidence of AID functionality within synovial tissue, we demonstrated that AID expression supports the expression of Iγ-Cμ circular transcripts in the RA synovial grafts, and hence the presence of ongoing class-switching from IgM to IgG. Because circular transcripts are only transiently produced in association with CSR [50], this demonstrates that class-switching is still ongoing within the RA synovial grafts, a result in line with the detection of human IgG ACPA in the mouse circulation. It is important to emphasise that these experiments cannot formally exclude the possibility that pre-existing long-lived plasma cells, either generated in situ or migrated from secondary lymphoid organs, might also contribute to the production of ACPA in RA synovium and in transplanted animals. Despite this limitation, the topographical proximity of ACPA-producing plasma cells to AID+ follicular structures and the close association between the level of ACPA production in the mouse serum and higher levels of AID within the same tissue strongly suggest that autoreactive plasma cells can be generated within ectopic lymphoid tissue. This concept is in agreement with the significantly higher levels of synovial ACPA recently demonstrated in synovial tissues containing lymphoid aggregates [55]. The lack of detection of GC CD20+ B cells reacting with citrullinated fibrinogen is in line with previous reports showing that reactivity toward labelled autoantigens is typically restricted to plasma cells surrounding ectopic GCs [43,56], and is in all probability due to the low density of antigen-specific antibodies on the cell surface of CD20+ B cells compared to the much higher quantity of high-affinity antibodies produced by plasma cells as they migrate out of GC. Finally, although the evidence that we provide is highly suggestive of a direct role for AID+ lymphoid structures in generating in situ autoantibody-producing cells, a formal demonstration would require evidence, i.e., in the SCID-HuRA model, that disruption of FDC networks abrogates plasma cell differentiation and ACPA production.

A final and highly relevant observation obtained in the HuRA-SCID chimera is that the RA synovium, in the presence of follicular structures expressing AID and CD21L, behaves as a self-sustained microanatomical unit of ectopic lymphoid tissue. Furthermore, within the time frame analysed, the RA synovium supports the proliferation of B cells and maintains an intrinsic ability to function as an autoantibody-producing site independent of circulating immune cells. The continued expression of AID and CD21L and the association with ongoing CSR and ACPA production in this environment support a key role for ectopic lymphoid tissue within the synovial membrane in driving the ongoing disease process, and may also explain the lack of direct correlation between peripheral B cell depletion and clinical response to the anti-CD20 monoclonal antibody rituximab [57,58]. The persistence of ectopic lymphoid structures in RA synovial tissues following transplantation into SCID mice is likely to be related to the sustained overexpression of genes regulating ectopic lymphoneogenesis and autoreactive B cell survival, such as the TNF family members LTβ, TNFα, and APRIL, as well as the lymphoid chemokine CXCL13 [26,27]. LTβ, TNFα, and CXCL13 were all significantly more highly expressed in AID+/CD21L+ as compared to AID−/CD21L− RA tissues, and high expression was maintained in RA synovial grafts displaying AID, ongoing CSR, and ACPA production. We suggest that the up-regulation of these key cytokines within RA synovial tissue allows differentiation and maintenance of FDC networks within T/B cell aggregates to form functional microanatomical immunological units, and hence, allow the up-regulation of AID and subsequent production of ACPA. In addition, our results might also explain the fall in ACPA levels resulting from the dual blockade of TNFα and LTα with etanercept [59], and the blockade of TNFα by infliximab [11] in patients with RA.

In conclusion, our work demonstrates that ectopic GC-like structures are not only functional in rheumatoid synovitis, but that their presence may contribute to disease pathogenesis via the production of ACPA. These data elucidate the mechanism of production of ACPA in the synovial membrane and thereby provide evidence of a pivotal role for AID in the pathogenesis of RA. We therefore propose that AID may be targeted for the development of novel therapeutic agents.

## Supporting Information

Alternative Language Abstract S1 Chinese translation of the abstract by Yvonne Ngar Woon Kam (30 KB DOC)

Alternative Language Abstract S2 German translation of the abstract by Fabia Brentano (32 KB DOC)

Alternative Language Abstract S3 Italian translation of the abstract by Michele Bombardieri (34 KB DOC)

## Abbreviations

Ab: antibody
ACPA: anti-citrullinated protein/peptide antibodies
AID: activation-induced cytidine deaminase
anti-CCP: anti-cyclic-citrullinated peptide
CD21L: CD21–long isoform
CFb: citrullinated fibrinogen
CSR: class switch recombination
Fb: fibrinogen
FDC: follicular dendritic cells
GC: germinal centre
HuRA-SCID: human RA-SCID mouse chimera model
IF: interfollicular
IHC: immunohistochemistry
QT-PCR: quantitative Taqman real-time PCR
SHM: somatic hypermutation
