# Supplementary material for: Ectopic Lymphoid Structures Support Ongoing Production of Class-Switched Autoantibodies in Rheumatoid Synovium
Source: PLoS Med. 2009 Jan 13;6(1):e1. doi: 10.1371/journal.pmed.0060001 (PMC2621263; doi:10.1371/journal.pmed.0060001)
Supplement: Supplementary file 1 [file pmed.0060001.sd001.doc]

**摘要**

**背景**

以滤泡树突细胞（follicular dendritic cell, FDC）网络为特征及类似生发中心（germinal center）的滤泡结构，已早被认为存在于自身免疫性疾病（autoimmune disease）的慢性发炎组织中。当中包括类风湿性关节炎（rheumatoid arthritis, RA）中的滑膜（synovium）。但到目前为止，这些异常结构会否驱使致病性自身抗体（autoantibodies）的形成，从而促进自身免疫及慢性发炎（chronic inflammation），仍处于争论中。抗瓜氨酸蛋白/肽抗体（anti-citrullinated protein / peptide antibodies, ACPA）是RA中非常特有的标记，因此被认定为此病的致病性及能够用以评估恶化预后。因此本研究的主要目的是检测RA中异常的淋巴结构是否：（一）具有激活诱导胞啶脱氨酶（activation-induced cytidine deaminase, AID），一种RNA编辑酶从而导致细胞高频率突变（somatic hypermutation）及类别转换重组过程（class-switch recombination, CSR）；（二）能维持持久性的CSR及诱发ACPA的形成，及（三）于毫无新免疫细胞汇入滑膜中的免疫缺陷小鼠　（SCID）／类风湿嵌合体（RA/SCID chimera）模型中持续着其机能性。

**方法及结果**

运用免疫组织化学染色法（immunohistochemistry）及即时定量聚合酶链锁反应器（Real-time PCR, QT-PCR）等方式于５５名RA患者的滑膜组织上，我们展示出AID总是表现在FDC^+^的结构中。而其分布与次要淋巴器官（secondary lymphoid organs）相似。

其次，对瓜氨酸纤维蛋白质（citrullinated fibrinogen）的免疫反应中展示了ACPA^+^/CD138^+^浆细胞（plasma cell）被AID^+^/CD21^+^ 滤泡结构包围。此外，我们认别出在生发中心以外的一种新颖的滑膜CD20^+^/AID^+^ B细胞亚群与滤泡间型大B细胞（interfollicular large B cell）相似。为了获取直接的证据来指出AID^+^结构能维持的CSR及于原位中进行ACPA类别转换，RA患者的滑膜组织被移植入34只SCID小鼠内。小鼠于移植４星期后被杀以便取出移植物及其血清。结果，我们发现表达着CD21L/FDCs的滑膜移植物中持续呈现AID及I_γ_-C_μ_环状转录物（circular transcript）(标记着持久IgM转换至IgG的生产过程)。另外，我们发现滑膜中的AID　mRNA水平与小鼠血清内循环中的人类IgG　ACPA有着紧密地联系。最后，我们也证明了调控异常淋巴新生（ectopic lymphoneogenesis）的基因持续高度性呈现，这与B细胞的存活及增生有着一定的联系。

**结论**

在这篇研究中，我们探究出以FDC^+^ 的滤泡结构往往呈现着AID，并被生产着ACPA的浆细胞围绕着。因此我们能提供有力的证据证明RA滑膜中异常的淋巴结构不但能发挥功能，而且能维持自身抗体的生产。其次运用具有生发中心的滑膜组织移植入SCID小鼠内，并在毫无新B细胞汇入的情况下驱使了人类IgG　ACPA 的形成。另外，加上持久呈现的AID、B细胞的增生以及持续CSR进行等的现象，加以肯定了先前我们定下的概念。因此本实验结果表明AID是一种有潜质的RA治疗性目标，同时也提出有机能的B细胞存活在滑膜中可能对慢性发炎自身免疫性疾病以及因B细胞不足治疗的反应有着一定的深远影响。
