# Supplementary material for: Ectopic Lymphoid Structures Support Ongoing Production of Class-Switched Autoantibodies in Rheumatoid Synovium
Source: PLoS Med. 2009 Jan 13;6(1):e1. doi: 10.1371/journal.pmed.0060001 (PMC2621263; doi:10.1371/journal.pmed.0060001)
Supplement: Supplementary file 2 [file pmed.0060001.sd002.doc]

**Hintergrund:**

Keimzentrum analoge follikuläre Strukturen, die durch ein Netzwerk von follikulären dendritischen Zellen charakterisiert sind, kommen in chronisch entzündeten Geweben bei verschiedenen Autoimmunerkrankungen vor, so auch in der synovialen Membran von Patienten mit rheumatoider Arthritis (RA). Es wurde jedoch lange debattiert, ob solche ektopischen Strukturen die Produktion von pathogenen Autoantikörpern antreiben und Autoimmunität und chronische Entzündungen fördern können.  Anti- Cyclische Citrullin Peptid-Antikörper (ACPA) sind hoch spezifische Marker für RA und haben einen hohen prognostischen Wert. Deshalb waren die Hauptziele der Studie zu bestimmen, ob ektopische lymphoide Strukturen im RA Synovium i) die „activation-induced cytidine deaminase (AID)“ exprimieren, das Enzym welches für somatische Hypermutation und Class-Switch Rekombination (CSR) der Immunoglobulin Gene verantwortlich ist, ii) die laufende CSR und die Produktion von ACPA unterstützen und iii) in einem Maus-Model (RA/SCID Chimera) funktionell aktiv bleiben, welches frei von einem Einstrom von neuen Immunzellen in das Synovium ist.

**Methoden und Resultate:**

Durch Immunhistochemie (IHC) und Taqman Real time PCR (QT-PCR) von synovialem Gewebe von einem total von 55 RA Patienten konnten wir sowohl auf mRNA wie auch auf Protein Ebene zeigen, dass FDC+ follikuläre Strukturen unverändert AID exprimieren und die Verteilung derjenigen in sekundären lymphoiden Organen weitgehend entspricht. Ausserdem wurde durch Immunreaktivität gegen citrulliniertes Fibrinogen gezeigt, dass AID+/CD21+ follikuläre Strukturen von ACPA+/CD138+ Plasmazellen umgeben sind. Zusätzlich identifizierten wir eine neue Untergruppe von synovialen CD20+/AID+ grossen B Zellen ausserhalb der Keimzentren, welche der kürzlich beschriebenen Population von interfollikulären grossen B Zellen ähneln. Um einen direkten Beweis zu erbringen, dass AID+ follikuläre Strukturen eine anhaltende CSR und auch die in situ Produktion von ACPA unterstützen, wurden 34 SCID Mäuse mit humanem RA synovium (Hu-RA SCID chimera) transplantiert und nach 4 Wochen wurden die Tiere geopfert um die humanen Transplantate und Maus-Sera zu analysieren. Die anhaltende Expression von AID und die Iγ-Cμ zirkulären Transkripte (Marker für den laufenden Isotyp Wechsel von IgM zu IgG) wurden durch IHC und QT-PCR in den synovialen Transplantaten die CD21L exprimieren beobachtet. Die Stärke der AID mRNA Expression in den Transplantaten war eng mit der Konzentration von zirkulierenden IgG ACPA im Maus-Serum assoziiert. Schliesslich waren das Überleben und die Zellproliferation von funktionellen B-Zell Nischen mit der anhaltenden Expression von Genen, welche die ektopische Lymphoneogenese regulieren, eng verbunden.

**Schlussfolgerung**

In diesem Bericht erbringen wir erstmals den Nachweis, dass ektopische lymphoide Strukturen im RA Synovium funktionell sind und die Autoantikörper-Produktion unterstützen. Dies, indem wir zeigen, dass follikuläre Einheiten, welche durch FDC Netwerke charakterisiert sind, unverändert AID exprimieren und von ACPA produzierenden Plasmazellen umgeben sind. Dieses Konzept ist zudem durch die anhaltende AID Expression, B-Zell Proliferation, fortwährende CSR und durch die Detektion im Maus-Serum von humanen IgG ACPA, welche von transplantierten Keimzentrum-positiven synovialem Gewebe abstammen. Diese Daten identifizieren AID als potentielles therapeutisches Ziel in RA und lassen vermuten, dass das Überleben von funktionellen B-Zell Nischen im rheumatoiden Synovium einen starken Einfluss auf die Modulation einer chronischer Entzündung und Autoimmunität hat und auch eine Antwort auf B-Zell verringernde Therapien ist.
