# Supplementary material for: Ectopic Lymphoid Structures Support Ongoing Production of Class-Switched Autoantibodies in Rheumatoid Synovium
Source: PLoS Med. 2009 Jan 13;6(1):e1. doi: 10.1371/journal.pmed.0060001 (PMC2621263; doi:10.1371/journal.pmed.0060001)
Supplement: Supplementary file 3 [file pmed.0060001.sd003.doc]

**ABSTRACT**

***Introduzione***

La presenza di strutture follicolari simili a centri germinativi (CG), caratterizzate da networks di cellule follicolari dendritiche (FDC), e’ stata a lungo descritta nei tessuti sede di infiammazione cronica in numerose malattie autoimmuni, tra le quali la membrana sinoviale in corso di artrite reumatoide (AR). E’ tuttavia fonte di controversia qualora tali strutture ectopiche contribuiscano all’autoimmunita’ e all’infiammazione cronica inducendo la produzione di autoanticorpi. Gli anticorpi anti-peptidi/proteine citrullinate (ACPA) sono considerati un marker altamente specifico di AR, predicono una prognosi peggiore ed e’ stato suggerito che svolgano un ruolo patogenetico. Per tale motivo, il principale scopo dello studio e’ stato quello di determinare qualora le strutture linfoidi ectopiche: i) esprimano l’enzima citidina deaminasi indotta dall’attivazione (AID), responsabile di mutazioni ipersomatiche e switch di classe dei geni delle Ig, ii) siano in grado di supportare lo switch di classe e la produzione di ACPA ad alta affinita’ e iii) rimangano funzionali e persistano in un modello animale (RA/SCID chimera) in assenza di un flusso continuo di cellule immunitarie nella sinovia.

***Metodi e Risultati***

Attraverso metodiche di immunoistochimica e Taqman Real-time PCR (QT-PCR) su tessuto sinoviale di 55 pazienti con AR, e’ stato possible dimostrare che strutture follicolari con networks di FDC esprimono invariabilmente AID con una distribuzione simile agli organi linfatici secondari. In aggiunta, mediante valuatazione dell’immunoreattivita’ verso il fibrinogeno citrullinato, abbiamo dimostrato come plasmacellule ACPA+/CD138+ si localizzino intorno a strutture follicolari AID+/CD21L+. Infine, abbiamo descritto per la prima volta nella sinovia un subset di linfociti B CD20+ che esprimono AID al di fuori dei centri germinativi e con caratteristiche simili alla popolazione di grandi cellule B interfollicolari. Al fine di ottenere un’evidenza funzionale diretta che le strutture follicolari AID+ siano in grado di supportare switch di classe e la produzione in situ di IgG ACPA, 34 topi SCID sono stati trapiantati con membrane sinoviali di AR e sacrificati dopo 4 settimane per la valuatazione sierologica e dei trapianti. Una persistente espressione di AID e dei trascritti Iγ-Cμ (in grado di identificare uno switch di classe IgM-IgG recente o in corso) e’ stata osservata nei trapianti sinoviali che esprimevano FDC/CD21L. In aggiunta, i livelli di espressione del trascritto di AID nella sinovia erano strettamente associati a quello degli ACPA IgG circolanti nel siero murino. Infine, la presenza di siti funzionali di proliferazione B linfocitaria era associata alla persistente e aumentata espressione di geni in grado di regolare la linfoneogenesi ectopica

***Conclusioni***

Questo studio, dimostrando che strutture follicolari con FDC esprimono invariabilmente AID e sono circondate da plasmacellule ACPA+, fornisce la dimostrazione originale che le strutture linfoidi ectopiche nella membrana sinoviale di AR sono funzionali e in grado di supportare la produzione di autoanticorpi. Tale evidenza e’ ulteriormente confermata dalla dimostrazione che, nel modello RA/SCID, trapianti di tessuti sinoviali con CG ectopici sono in grado di sostenere l’espressione di AID, la proliferazione B cellulare, lo switch di classe e la produzione di ACPA umani IgG in assenza di un nuovo influsso di cellule B dal circolo sistemico. Questi risultati identificano in AID un nuovo potenziale target terapeutico nell’AR e suggeriscono che la sopravvivenza di siti funzionali di cellule B nella sinovia e’ in grado di influenzare in modo profondo l’infiammazione cronica, l’autoimmunita’ e la risposta a farmaci biologici che provocano la delezione B.

**TRANSLATION BY:** Michele Bombardieri
